# The Many Ways of Experiencing Solitude: Personality Processes, in Context, as Predictors of Time Alone

**DOI:** 10.1111/jopy.12968

**Published:** 2024-08-12

**Authors:** Netta Weinstein, Mark Adams

**Affiliations:** ^1^ School of Psychology and Clinical Language Sciences University of Reading Reading UK

## Abstract

This article integrates insights from the Journal of Personality's Registered Report‐only special issue, which explores the relationship between personality and experiences within solitude. Contrary to the traditional view that solitude primarily serves those who are introverted or seeking refuge from social interactions, findings in this issue demonstrate that solitude is actively sought by, and may hold benefits for, a broad spectrum of personality types. We discuss these findings and suggest there may be more complex interactions between personality and solitude than previously recognized. We highlight the importance of conceptual and methodological clarity in studying both personality and solitude. Studies also show that the benefits of solitude for well‐being depend on contextual factors including the function and purpose of solitude, and activities undertaken (or not) when alone. Preferences for, and enjoyment of, solitude are influenced by more than just personality traits; they are shaped by how personality interacts with specific situations and contexts. We provide practical recommendations for future research to refine methods in order to better understand the nuanced experiences of solitude. These approaches will help clarify the conditions under which solitude is most beneficial and offer deeper insights into how solitude can improve well‐being for different individuals.

## 1 Introduction

Personality psychology has largely focused on individual differences that are shaped by, and shape, the social context. However, solitude—the state of being alone and not in direct social contact—is a significant aspect of our daily lives, and individual differences can affect how much people need or avoid, and how they experience, solitude. On average, adults spend about one‐third of their waking time in solitude, a figure that increases to nearly half for older adults (Livingston 2019). The emotions experienced in solitude vary widely, ranging from emptiness and loneliness to sensations of peace and awe (Coplan, Zelenski, and Bowker 2017; Long and Averill 2003; Mateer 2022; Nguyen, Ryan, and Deci 2018; Thomas 2023). What factors determine how solitude is experienced? How do individual differences influence people's need for, aversion to, and well‐being in solitude? The empirical study of solitude as either detrimental (e.g., lonely) or beneficial (e.g., peaceful) is still in its early stages, but the field of personality psychology has much to offer in enhancing our understanding of it.

In exploring this topic, research must be fit‐for‐purpose for the study of solitude as a standalone experience. At present, the notion that solitude is linked to specific personality types is grounded in the understanding that certain individuals are drawn to, and thrive in, particular social situations. It might stand to reason that the same personality qualities also explain why some people are drawn to, and thrive in, solitude, as the “other side of the coin” to the social world. However, solitude is a distinct and powerful everyday context in its own right (Storr 2015), and researchers should not assume that similar (or perhaps opposing) personality processes will drive solitude and social experiences alike.

The articles in this issue examine theory‐informed models of personality in relation to solitude with studies that were peer‐reviewed by experts in the area, registered before data collection in most cases, and before analysis in all cases. Unlike replication‐focused registered reports, which aim to confirm or disconfirm established research findings (e.g., Brandt et al. 2014), these early‐stage, innovative studies are guided as much or more by theory drawn from personality psychology and early conceptualizations within the emerging field of solitude. Consequently, the studies often produce surprising or non‐significant findings. Overall, the findings—both expected and unexpected—offer important insights into the growing body of solitude research, informing future conceptual and methodological decisions within studies that predict solitudinal experiences. We review these insights below and discuss opportunities for future research to better understand the role of personality in solitude.

## 2 Lessons Learned About the Study of Personality in Relation to Solitude

### 2.1 Lesson 1: Conceptual Clarity Is Needed to Identify the Solitude‐Loving Personality

The first lesson we might draw from articles within this issue is that personality processes in relation to solitude are complex, dependent on the measures selected to operationalize them, and that they may counter intuitions about solitude. Researchers examining personality in relation to solitude must, therefore, carefully determine how the personality processes they study should be conceptualized and measured with solitude‐specific mechanisms in mind. For example, Thomas (2024) explores the relations between introversion and motivation for, as well as time spent in, solitude, comparing aspects of introversion that might drive the relationship with solitude. The conceptual links between introversion and solitude are intuitive. Introverts, who are often described as gaining energy from time spent alone (e.g., Helgoe 2013; Janssen 2021), are thought to both prefer and benefit from solitude. However, Thomas (2024) finds surprising results when using the most common measure of introversion—the Big‐Five Inventory (John, Donahue, and Kentle 1991; John and Srivastava 1999), which defines introversion (in a nearly tautological way to solitude‐seeking itself) as having lower levels of sociability, assertiveness, and activity, and higher levels of reserved, reflective, and solitary behavior (John and Srivastava 1999). Contrary to expectations, Thomas (2024) observes that introverted individuals have *lower*, not higher, autonomous—choiceful and volitional—motivation for solitude and spend less, not more, time in solitude. Similarly, in past research, Nguyen, Weinstein, and Ryan (2022) found no relation between the Big‐Five Inventory and motivation for solitude. On the other hand, in the current issue, Nguyen, Konu, and Forbes (2024) observe that individuals high in extraversion report being in solitude more frequently than individuals lower in extraversion. In all, findings concerning the Introversion‐Extraversion dimension of the Big‐Five seem mixed, but using traditional measures of the construct, there is little consistency in evidence that introverts are drawn to solitude as might be intuited.

Conversely, using a more nuanced measure of introversion (the multi‐dimensional STAR scale; Cheek, Brown, and Grimes 2014), Thomas (2024) reveals a different pattern. Aspects of introversion titled “social introversion”—a preference for being alone—and “thinking introversion”—a tendency to engage in deep, reflective thought and to enjoy being immersed in one's inner world—are, as expected, associated with a desire to spend time alone and with spending more time regularly in solitude. This research makes evident the need for a more precise conceptualization of the construct of introversion, particularly where it is linked to solitude. While certain qualities of introversion may predispose individuals to seek and benefit from solitude, clarity in both concept and measurement are essential to capture these associations.

In identifying that *thinking* introverts—those who self‐reflect—value and spend time in solitude, Thomas (2024) taps into a cluster of individual differences that might be especially important in the study of solitude. Specifically, it may be that dispositions towards curiosity, interested self‐reflection, and self‐congruence (acting in ways consistent with the self) predispose individuals to benefit from solitude. Similar findings have been observed following qualitative interviews conducted about positive solitude experiences; namely, that those who are curious prefer and experience well‐being in solitude (Weinstein, Hansen, and Nguyen 2023a). Furthermore, diary studies have suggested those who are autonomously oriented (i.e., self‐interested, self‐congruent, and unlikely to act from external pressure) pursue solitude for its perceived value (Nguyen, Weinstein, and Ryan 2022).

Also tapping into this personality profile, another article within this issue identifies that an *autotelic* personality—one characterized by curiosity, challenge seeking, and freedom from pressure (Tse et al. 2020)—is linked with more flow in solitude. Autotelic propensities may even influence pathways between more established personality traits and solitude outcomes. For example, additional findings in this study suggest that the relationship between extraversion and the experience of solitude does not exhibit the anticipated negative linear trend; rather, extraversion interacts with autotelic personality to achieve a flow state in solitude (Tse, Joseph, and Sweeny 2024). Taken together, these findings challenge the common assumption that solitude is predominantly for individuals predisposed towards shyness, anxiety, or those seeking refuge from social demands. They suggest that personality oriented towards reflection, autonomy, and curiosity may be essential antecedents of solitude seeking and enjoyment.

### 2.2 Lesson 2: Personality Effects Depend on the Functions of Solitude and Social Time

A second lesson we might draw is that researchers studying the role of personality in solitude should be sensitive to interactions between personality and the particular characteristics of the specific solitude moments under study because main effects of personality may heavily depend on those situation‐specific characteristics. Articles particularly draw attention to the possibility that solitude has multiple distinct *functions*, which may serve the needs of people differently based on their individual differences. For example, Nguyen, Konu, and Forbes (2024) find that people having high‐arousal negative emotions (such as anxiety) prefer to be in solitude, whereas those who had high‐arousal positive emotions (such as excitement) prefer to be with others. Presumably, solitude is preferred *because* it offered a space to downregulate those high‐arousal negative emotions, a benefit observed in earlier research (Nguyen, Ryan, and Deci 2018). In addition, personality predictors of experiences in solitude depend on those functions. Individuals with different characteristics, and therefore different needs, use solitude differently to gain well‐being benefits from this time.

Personality by context interactions have high explanatory power. Indeed, Ren et al. (2024) find that in predicting perceived importance of solitude to individuals, Big‐Five personality traits account for about 22% of variance, but an even greater percentage of variance (46%) in solitude importance can be accounted for by interactions between personality and specific solitude functions. The authors identify, for example, that those higher in neuroticism place importance on the emotion‐regulation function of solitude, while those lower in extraversion (i.e., introverts) pursue solitude to feel calm. Importantly, this research also helps to explain mixed findings regarding the introversion‐extraversion distinction and solitude seeking: Extraverts do not find solitude to be *less* important, rather they find it to be important for different reasons.

On the flip side, solitude might be seen as a *barrier* to functions provided by the social context, in which case people may disprefer to be alone. Zeigler‐Hill, Vonk, and Fatfouta (2024) examine this possibility by measuring solitude *avoidance* as a function of distinct qualities of narcissism, those who take advantage of others from a place of entitlement and grandiosity (Morf and Rhodewalt 2001). In a series of studies, the authors examine various forms of narcissistic personality traits and find that extraverted narcissists—those who seek others' attention and esteem—disprefer solitude when their status was high. For these individuals, social interaction served a purpose that solitude disrupts. Together, this research suggests that personality can be brought to both solitude and social contexts to understand which functions each context holds for people with specific individual differences. When the functions of solitude are salient, social time may be relatively less appealing, and vice versa—when social interactions serve an important function, solitude time is felt to be aversive. In all, personality processes appear to dispose individuals to pursuing distinct functions of both social and solitude contexts.

### 2.3 Lesson 3: Inspiring Approach Motives May Be Key to Positive Solitude Experiences

Beyond what solitude can and cannot *do* for us, articles in this issue highlight that the way we *approach* solitude may be important for determining how we experience it. It is an intriguing area of study because multiple constructs that reflect one being *drawn* to solitude produce diametrically different outcomes. Researchers have previously suggested that preference of solitude may be dysfunctional when it reflects avoidance motivation (Nguyen, Weinstein, and Ryan 2021). In this issue, Huang et al. (2023) investigate this possibility through analyzing neurophysiological patterns associated with solitude to reflect underlying motivational states. The authors find that preference for solitude is associated with avoidance motivation, indicated by increased right frontal alpha asymmetry and decreased left frontal alpha asymmetry—markers typically linked with avoidance motivation (Wendel, Wilhelm, and Gable 2021).

Whereas Huang et al.'s (2023) findings suggest that preferring solitude might be an indicator of dysfunction that reflects *avoiding* social contexts rather than approaching (i.e., pursuing) solitudinal ones, previous studies have highlighted the two—avoidance and approach—may be better understood as distinct motives rather than two ends of a single motive continuum (Elliot 2006). In a separate article within this issue, Rodriguez et al. (2023) suggest that if people can be encouraged to hold *positive expectations* that lead them to pursue solitude actively—rather than preferring merely to avoid social interactions—they can gain affective benefits from being alone. In examining the potential benefits of solitude, Rodriguez et al. (2023) find that positive reappraisal of solitude significantly enhances low‐arousal positive affect in lonely individuals after a 10‐min solitude session, compared to a control group where no reappraisal occurred. Together, these studies highlight the importance of delineating between preferences for solitude that are approach‐oriented, and those that are avoidance motivated to better understand how motives drive the solitude experience. Furthermore, they point to a need to further explore strategies to help inspire individuals' approach‐oriented pursuit of solitude, perhaps through mindsets, as did Rodriguez et al. (2023, and earlier in 2020), or through other strategies that activate people to engage in their solitudinal moments.

### 2.4 Lesson 4: Being May Not Be Better than Doing. There May Be a Role for Both

Articles within this issue also speak to two ways of experiencing solitude that have been distinguished in a past theoretical article; being (i.e., existing, in thought, or being ‘in the moment’) and doing (goal‐oriented actions; Littman‐Ovadia 2019). Examining both, McVarnock et al. (2023) measure the variety and importance of activities selected by emerging adults who report how they typically spend time in solitude. Their findings indicate a range of activities that emerging adults undertake, including active “doing” pursuits, such as watching Netflix, passive social media use, or doing chores or homework, and passive “thinking” based activities such as daydreaming or planning. The authors find that “thinking” based solitude (i.e., “Pure” or “total” solitude; Weinstein, Hansen, and Nguyen 2023b) is associated with greater loneliness, whereas “doing” based solitude is associated with lower *aloneliness* (the feeling of needing time alone) and is not associated with loneliness. This suggests, at least for emerging adults, passive, unstructured solitude activities were unpleasant and unrewarding. Similarly, Tse, Joseph, and Sweeny (2024) observe benefits when participants engage in a mildly absorbing task, suggesting that active solitary pursuits can also lead to measurable positive changes in well‐being. Together with a previous study (e.g., Wilson et al. 2014) that found people often reported sitting with their thoughts, even for a brief period, as aversive and unpleasant, this work indicates that the quality of solitary activities plays a crucial role in how time spent alone is perceived. It is not simply the amount of time spent in solitude that matters, but the *quality* of the solitude time and how it is spent that is important.

In contrast, work by Bradshaw, Ferber, and Ryan (2024) in this issue highlight a place for ‘being.’ The authors examine the influence of memories recalled close to entering solitude on emotional well‐being during time spent alone. Participants recalled intrinsic versus non‐intrinsic (Study 1) or extrinsic (Study 2) goal accomplishments prior to spending five minutes alone in solitude. Their studies produce two key findings worth mentioning here. First, that even an extremely brief period of ‘being’ (activity‐less) solitude is sufficient to elicit significant post‐solitude well‐being improvements. Second, these improvements are consistent across all memory conditions, indicating that the well‐being benefits a robust, though not contingent upon the type of memory engaged in during solitude. In other words, this evidence suggests that solitude can serve as replenishing time, whether approached with a positive, enriching mentality or at least a neutral, benign frame of mind. Are these different findings due to some studies relying on *brief* periods of solitude where others rely on everyday and often lengthier solitude periods? It may be that moderate amounts of *complete* solitude characterized by ‘being’ (i.e., fully alone and sitting with one's own thoughts, Weinstein, Hansen, and Nguyen 2023b) conduce well‐being, whereas longer such time spans benefit from having some meaningful and rewarding activities. But it may also be that certain individual differences, including personality or age (e.g., younger or older adults), affect whether activities or reflection conduce well‐being in solitude.

## 3 Future Directions

### 3.1 Future Directions in Personality Antecedents of Solitude Experiences

The articles within this special issue point to important next steps in the study of solitude. Building on their efforts, future robust research efforts are needed to refine the conceptualization and measurement of personality, and specifically, introversion and extraversion, in the context of solitude. Are people drawn to solitude because they simply have a disposition or tendency towards time alone, or do individual differences that reflect a relationship with the *self—*such as self‐reflection, curiosity, and self‐connection—drive solitude pursuit and benefit? To date, qualitative studies have suggested these aspects of personality may predispose individuals to seeking and enjoying solitude because they see the *value* in time alone (Weinstein, Hansen, and Nguyen 2023a). Through exploring the autotelic personality (Tse, Joseph, and Sweeny 2024), thinking introversion (Thomas 2024), and even mindfulness (Bradshaw, Ferber, and Ryan 2024), articles within this issue find some empirical support for these views. However, more work is needed to identify robust personality predictors with clear mechanisms for their solitude benefits.

In posing these questions, both the conceptualization and the measurement of personality matter. The studies in this issue reveal that traditional measures, such as the Big‐Five Inventory, provide mixed results regarding the link between introversion and solitude. However, more nuanced scales, like the STAR scale, show that specific aspects of introversion, such as social and thinking introversion, are associated with a desire for solitude. These distinctions reflect a growing focus within psychology to be cautious in both defining and operationalizing measures with concerns about jingle–jangle fallacies (wherein aspects of different scales ostensibly measuring the same construct, have different definitions and items; or where scales with different labels have, in effect, identical definitions and items) in personality psychology and psychology more broadly. This issue undermines our trust in conceptual insights drawn from work without deep consideration of these problems (Altgassen, Geiger, and Wilhelm 2024; Irwing et al. 2024; Wulff and Mata 2023; Ziegler, Booth, and Bensch 2013). Further research is needed to develop and consider these nuanced measures in relation to one another to better understand the complex relationship between personality traits and solitude preferences.

In addition, the articles in this issue reveal that personality, context, and attitude each significantly impact the solitude experience. More importantly, they demonstrate the need to explore the interactions among these factors to understand when, how, and for whom solitude benefits or undermines psychological well‐being. Solitude and social contexts may serve separate and distinct functions for well‐being (Luo et al. 2022; Thomas 2023), and studies that address both individual differences and specific solitude moments form the ideal platform for examining how those functions meet the different needs of individuals with different emotion‐regulation, self‐concept, or relational tendencies. Similarly, personality may both predict and moderate attitudes and motives towards solitude. For example, positive expectations people have for solitude may be influenced by social norms (Rodriguez et al. 2023), but also by underlying personality traits that shape people's ‘capacity to be alone’ (Winnicott 1958). These may tap into curiosity or self‐congruence (Tse, Joseph, and Sweeny 2024).

But how solitude meets expectations may depend at least in part on how well solitude functions to support people's needs. For example, in this issue Lay et al. (2024) show that individuals often overestimate both low‐arousal affective states such as feelings of calmness, relaxation, and loneliness, as well as positive and negative high‐arousal states like feeling energetic or irritated. Within this study, those who willingly choose to enter solitude (i.e., Self‐Determined Solitude) remember their experiences more positively, whereas those do not purposefully enter solitude (i.e., non‐self‐determined Solitude) overreport feelings of loneliness. The research suggests that people's memories of their emotional state do not always accurately reflect momentary reports of how they felt in situ. Rather, they may be influenced by biased priors when reflecting on past feelings in situations where affective information is no longer directly accessible. As such, future research examining well‐being experienced in solitude, recalled after solitude, and even residual—lasting beyond solitude moments to improve subsequent experiences within a day or cumulative experiences across longer spans of time—would greatly inform this area and our understanding of how solitude contributes to everyday life.

### 3.2 Conceptualizations of Solitude

To explore personality processes, studies in this issue relied on a broad range approaches and methods, reflecting the richness of approaches available to study solitude. For one, solitude is conceptualized and operationalized in diverse ways. Bradshaw, Ferber, and Ryan (2024), Huang et al. (2023), and Rodriguez et al. (2023) examine “pure” or “total” solitude (i.e., simply sitting with your thoughts), whereas McVarnock et al. (2023), Ren et al. (2024), Thomas (2024), Tse, Joseph, and Sweeny (2024), and Zeigler‐Hill, Vonk, and Fatfouta (2024) incorporate *doing*—engagement in activities—so long as individuals are physically separated and not interacting. Lay et al. (2024) and Nguyen, Konu, and Forbes (2024) broaden the definition of solitude to mean the lack of interaction with others (face‐to‐face or virtually) regardless of others' physical proximity. These definitions converge on several points. First, solitude is distinct from isolation and loneliness. Second, solitude entails a psychological state of the individual that necessitates not interacting with others (face‐to‐face or digitally). The range of these definitions even within a single journal issue highlights that we may not be able to rely on a singular “correct” definition of solitude. Instead, solitude is a multifaceted construct that encompasses not (only) the physical act of being alone but also the emotional and cognitive experiences that accompany it. An appreciation for this spectrum of solitude definitions is crucial as research in this area continues to evolve, and to ensure that our research findings are comparable and interpretable.

The concept of being “in solitude” raises important questions about both its definition and measurement. As we discussed in earlier sections, there are a range of definitions of solitude currently being used in the literature covering most, but not all, open questions. For instance, one immediate question remains: how do we differentiate solitude from simply “waiting” behavior? This question is especially pertinent to studies that use brief (i.e., 5 to 15 min) solitude tasks as their primary standard of solitude time, where distinguishing solitude from inactivity is crucial. This distinction is also important in research investigating “pure” or “total” solitude, where individuals are alone with just their thoughts. Waiting typically involves inactively counting down time until the next event or activity begins, often leading to feelings of impatience or boredom (Leung 2015; Wilson et al. 2014). In contrast, “pure” solitude involves active engagement in self‐reflection or inward focus. Both waiting and “pure” solitude involve unstructured passivity but differ fundamentally in their intent and mental engagement, making it vital to separate the two. Furthermore, there is a danger that we can turn “pure” solitude into mere “waiting” behavior with no outwardly observable difference in the participant; for example, by simply alerting the individual to how long they will spend alone. Misclassifying waiting behavior as “solitude” could easily skew results regarding potential benefits or attribute the source of these benefits incorrectly. For example, improvements in well‐being during solitude might be misattributed to solitude‐conducive internal aspects, such as deeper reflection and self‐connection (i.e., characteristics typically associated with “pure” solitude), when they are instead due to the reduction of external pressures like stress, environmental noise, and social burnout.

Working operationalizations of solitude become even more critical as research examines this concept across ever more varied contexts for being alone. Within the scope of this issue alone, the application of solitude (i.e., the solitude task) varies significantly, from brief, at‐home, periods of reflection in front of an online questionnaire to designated laboratory‐based interventions and detailed retrospective accounts of daily activities. Each of these contexts may preferentially align with certain definitions of solitude. The strength of future research in this area may lie not in adhering to a singular, unyielding definition of solitude, but rather in embracing a more open approach to reporting and disseminating our findings. Key to this will be a commitment to standardized ways of reporting exactly how solitude is defined in each study, accompanied by a clear explanation of the rationale for exploring certain aspects of solitude over others found in the literature.

Standardizing definitions allows researchers to switch between different understandings of solitude based on their specific research questions, even if it falls outside their usual focus. This flexibility opens new research opportunities while maintaining a cohesive view of how their work fits into the larger body of literature. Such an approach to conceptual and methodological transparency not only improves clarity but also promotes a richer, more collaborative exploration of solitude's dimensions, fostering a more inclusive and comprehensive academic dialogue. This practice is already evident in some articles in this issue, and we strongly encourage more researchers to adopt it going forward.

### 3.3 Classifying Benefits of Solitude

Throughout this issue, and in most recent literature examining the benefits of positive solitude, various measures of these purported benefits are naturally included. It is worth considering, however, what exactly constitutes a benefit. Typically, articles examining the benefits of solitude fall into one of three categories.

The first category includes studies that examine the immediate effects of solitude. These studies often report an upturn in mood and a corresponding decrease in negative mood upon completing a (usually brief) period of solitude. Typically used in lab‐based studies, this method involves participants completing pre‐and‐post measures of affect and is useful for investigating immediate or short‐term benefits of solitude. For example, Bradshaw, Ferber, and Ryan (2024) in this issue report improvements in mood after just five minutes of solitude. The second category involves longitudinal methods, such as diary studies or Experience Sampling Methods (ESM), where participants' moods, interactions, and solitude events are tracked over time. These studies provide a more dynamic view of solitude, showing how the benefits of solitude can unfold day by day, and within a day. The third category includes intervention‐based paradigms where participants engage in structured solitude activities. These interventions aim to reshape participants' attitudes towards solitude through a combination of reframing solitude more positively (e.g., Rodriguez, Bellet, and McNally 2020; Rodriguez et al. 2023) and providing structure for their solitary experiences (e.g., Adams and Weinstein 2023). This approach highlights how structured solitary time can positively influence mental health. Such studies are crucial for understanding how regular practice and exposure to solitude can alter both perceived and actual benefits. Furthermore, they can help identify strategies for making solitude a more positive and enriching experience.

However, despite their utility, the majority of current research methods share a critical limitation: they primarily focus on the benefits of solitude through the lens of immediate, short‐term emotional states. Most studies concentrate on solitude's capacity to enhance emotional well‐being and reduce negative affect shortly after a solitary experience. While important, these approaches overlook the potential for long‐term or sustained benefits of solitude that may not be immediately apparent but develop over time. Furthermore, there is an inherent bias in viewing immediate mood improvements from enjoying solitude as the standard to which we should consider solitude as beneficial. This is akin to judging the effectiveness of medicine based on how much one enjoys the taste of it. This bias is especially pronounced in studies that rely on participants' reports from single, short‐duration experiences of solitude, favoring those predisposed to enjoy solitude. Instead, there is good reason to believe that the benefits of solitude may accrue over time, and that repeated exposure to the short‐term benefits—such as increased peaceful mood and reduction of high‐arousal stressors—could lead to more substantial, compounded effects. Additionally, familiarity effects may be at play, where becoming accustomed to solitude could help reduce an individual's initial resistance, thereby making subsequent periods of solitude more enjoyable and beneficial. Future studies could benefit immensely from considering more longitudinal perspectives to fully explore how solitude can foster deeper, long‐term psychological benefits.

## 4 Conclusion

Solitude is a state sought out and valued, or alternatively avoided, by people with different personality profiles. We are just beginning to grasp which individual differences predict the draw to solitude, the capacity to be alone, and the propensity to benefit from solitude time. Building this understanding is important given the significant role individual differences play in shaping the considerable time people spend alone each day. Researchers studying personality and solitude would benefit from considering the functions that both solitudinal and social moments hold for individuals with different needs and challenges, as well as the motives and attitudes towards solitude held by different individuals. They should carefully conceptualize and measure both personality and solitude. Articles within this issue underscore the need to reconsider which are key personality traits in the context of solitude and to expand research beyond traditionally examined factors. Such an approach offers a deeper insight into the personal differences that affect one's desire for and enjoyment of being alone.

## Author Contributions

N.W. and M.A. co‐authored this manuscript.

## Conflicts of Interest

The authors declare no conflicts of interest.
